# A multi-epitope vaccine designed against blood-stage of malaria: an immunoinformatic and structural approach

**DOI:** 10.1038/s41598-022-15956-3

**Published:** 2022-07-08

**Authors:** Amir Atapour, Parisa Vosough, Somayeh Jafari, Gholamreza Anani Sarab

**Affiliations:** 1 Department of Medical Biotechnology, School of Advanced Medical Sciences and Technologies, Shiraz University of Medical Sciences, Shiraz, Iran; 2 Department of Molecular Medicine, School of Medicine, Birjand University of Medical Sciences, Birjand, Iran; 3 Cellular & Molecular Research Center, Birjand University of Medical Sciences, Birjand, Iran

**Keywords:** Biotechnology, Computational biology and bioinformatics, Microbiology

## Abstract

Malaria is a complex disease caused by parasites of the genus Plasmodium and is the leading cause of morbidity and mortality worldwide. The most severe form of malaria disease is caused by *Plasmodium falciparum*. Thus, a combination of different approaches is needed to control malaria. Resistance to first-line drugs and insecticides, on the other hand, makes the need for an effective vaccination more urgent than ever. Because erythrocyte parasites cause the most clinical symptoms, developing a vaccination for this stage of infection might be highly beneficial. In this research, we employed various bioinformatics methods to create an efficient multi-epitope vaccine that induces antibodies against the blood stage of malaria infection. For this purpose, we selected the malaria PfGARP protein as the target here. The B, HTL epitopes, and epitope conservation were predicted. The predicted epitopes (including 5 B and 5 HTL epitopes) were connected using suitable linkers, and the flagellin molecule was used as an adjuvant to improve its immunogenicity. The final construct vaccine with 414 amino acids long was designed. The vaccine's allergenicity, antigenicity, solubility, physicochemical characteristics, 2D and 3D structure modeling, molecular docking, molecular dynamics simulation, in silico cloning, and immunological simulation were tested. In silico immune simulation results showed significantly elevated IgG1 and IgM and T helper cells, INF γ, IL 2, and B-cell populations after the injection of the designed vaccine. These significant computational analyses indicated that our proposed vaccine candidate might activate suitable immune responses against malaria. However, in vitro and in vivo studies are essential for further validation.

## Introduction

Malaria is a devastating disease caused by a unicellular protozoan parasite transmitted to humans through the bites of infected mosquitoes^1^. A massive number of people worldwide afflict this disease. The World Health Organization (WHO) reported that malaria was responsible for about 627,000 deaths globally in the year 2020^2^. Six parasite species cause malaria in humans: *Plasmodium falciparum*, *Plasmodium vivax*, *Plasmodium malariae*, *Plasmodium ovale curtisi, Plasmodium ovale wallikeri, *and *Plasmodium Knowle*^3^. People heavily exposed to mosquito bites infected with *P. falciparum* acquire a more severe type of malaria and have a higher chance of death. worse of all, *falciparum* has acquired resistance to nearly all antimalarial medications currently available^4^. The high level of *falciparum* resistance to chloroquine (CQ) and then to sulfadoxine-pyrimethamine (SP) has created the need for newer and more effective treatments such as vaccines^5^. Genetic, antigenic diversity and the ability to evade host immune responses are major obstacles hindering the development of effective anti-malaria vaccines. Many studies have focused on conserved regions or epitopes common between vast ranges of plasmodium strains^6^. *P. falciparum* glutamic acid-rich protein (PfGARP) is an 80 kDa antigen expressed on the surface of infected erythrocytes with *P. falciparum*^7^. Remarkably, the PfGARP gene is absent in other species of human malaria parasites and is expressed in early-to-late-trophozoite stages^8^. PfGARP is found on the periphery of infected erythrocytes, and whole-genome sequence data indicates that, despite its apparent immunogenicity, PfGARP is conserved without any deletions^9^. Studies demonstrated that PfGARP plays a functional role in enhancing the adhesive properties of human erythrocytes that are unique to *P. falciparum*–mediated pathogenesis^10^. Generally, the parasite's adhesive properties, a strategy used to avoid immune surveillance and splenic clearance of infected RBCs, contribute to virulence^11^. Interestingly, PfGARP has recently been identified as the target of protective antibodies. In the trophozoite stage, antibodies to PfGARP disrupted mitochondrial membrane integrity and induced programmed cell death of infected RBCs. Then, it will eventually lead to a decrease in acute malaria. Also, vaccination with PfGARP protects monkeys from being confronted with the malaria parasite^12,13^. Immunity against malaria parasites is complex and fundamentally stage- and species-dependent^14^. Many attempts have been made to develop a vaccine against malaria, but its complex multistage life cycle exhibits special challenges for vaccine development^15^. RTS, S, or Mosquirix is a recombinant protein-based vaccine, and the world's first malaria vaccine proved to provide partial malaria protection in young children. This vaccination avoids 39% of malaria cases and 30% of severe malaria cases. Despite considerable efforts, there are certain drawbacks. It is less effective, requires four doses, and gives less protection after a few months^16,17^. Malaria's blood stages are responsible for morbidity and mortality in humans. Therefore, vaccines that target these stages try to protect the human host from clinical disease and death^18^. According to the information presented above, the PFGARP antigen makes infected RBCs an attractive target for vaccine development, mainly because the parasite is immediately exposed to the host humoral immune response during the blood-stage. Immunogenicity of vaccinations can be increased by using adjuvants such as Toll-like receptor (TLR) agonists. TLRs are present in macrophages, dendritic cells (DCs), and specific epithelial cells to provoke potent and immediate innate immune responses, which lead to subsequent adaptive immune responses^19^. TLRs 1, 2, 4, 5, 6, and 10 are expressed on the cell surface, and TLR5 recognizes flagellin (fliC), a bacterial flagella component that inherently attaches to TLR5 as a natural agonist^20^. FliC protein is a highly conserved bacterial protein; it can stimulate the humoral and cellular immune system by producing antibodies, cytokines, and interleukins that prevent the parasite entry in the pre-erythrocytic stage of malaria^21–23^. Therefore, the present study aimed to design a multi-epitope vaccine by utilizing bioinformatics methods based on PfGARP epitopes to elicit a humoral immune response against blood-stage of malaria infection.

## Results

### Linear B-cell epitope prediction

Epitopes with 16 amino acid length in (PfGARP) protein were selected for use in the final construct of the vaccine. Five B-cell epitopes that had higher scores are shown in Table 1.

**Table 1:** List of the five B-cell epitopes from PfGARP protein by ABCpred Server.

Rank	Sequence	Start position	Score
1	HGEENLYEEMVSEINN	162	0.93
2	HETSNDTKDNDKENIS	204	0.91
3	AEEDDDDAEEDDDDAE	596	0.89
4	CEEQHITVESRPLSQP	441	0.89
5	GCGIISSVHETSNDTK	186	0.89

### MHC class II peptide-binding prediction (CD4+ T cell epitope)

MHC class II peptide binding epitopes with the highest score for human class II alleles were predicted using the IEDB MHC-II server. Then, the top predicted epitopes with high scores were only chosen for generating the final vaccine construct (Table 2).

**Table 2:** List of the top predicted MHC class II peptide from (PfGARP) protein, by IEDB MHC-II server.

Allele	HTL epitopes	Method	Percentile rank	Smm_ic50
2-16 HLA-DRB1*01:03	FSNGLLKNQNILNKS	NetMHCIIpan	20.00	N/A
234-248 HLA-DRB1*01:05	TLDKKERKQKEKEMK	NetMHCIIpan	100.00	N/A
91-105 HLA-DRB1*01:03	SVDKKKDKKEKKHKK	Consensus (comb.lib./smm/nn	100.00	N/A
401-415 HLA-DRB1*01:03	DKGKHKKAKKEKVKK	NetMHCIIpan	82.00	N/A
12-26 HLA-DPA1*01/DPB1*04:01	ILNKSFDSITGRLLN	Consensus (comb.lib./smm)	11.35	1257.00

### Conservancy analysis of epitopes

The sequence fraction containing each epitope was defined to calculate each epitope's conservancy level within the collected homologous sequence set; Table 3 summarizes the results. As shown in Table 3, epitopes are present in all homologous sequences with a conservancy degree from 20 to 100 percent. Epitope 7 is the most abundant (14 out of 20).

**Table 3:** Epitope conservancy degrees were analyzed using the tool (http://tools.iedb.org/conservancy).

Epitope	Epitope name	Epitope sequence	Epitope length	Percent of protein sequence matches at identity < = 100%	Minimum identity (%)	Maximum identity (%)
1	Epitope 1	HGEENLYEEMVSEINN	16	55.00% (11/20)	31.25	100.00
2	Epitope 2	HETSNDTKDNDKENIS	16	55.00% (11/20)	31.25	100.00
3	Epitope 3	AEEDDDDAEEDDDDAE	16	30.00% (6/20)	25.00	100.00
4	Epitope 4	CEEQHITVESRPLSQP	16	55.00% (11/20)	18.75	100.00
5	Epitope 5	GCGIISSVHETSNDTK	16	45.00% (9/20)	25.00	100.00
6	Epitope 6	FSNGLLKNQNILNKS	15	75.00% (15/20)	26.67	100.00
7	Epitope 7	TLDKKERKQKEKEMK	15	70.00% (14/20)	40.00	100.00
8	Epitope 8	SVDKKKDKKEKKHKK	15	45.00% (9/20)	46.67	100.00
9	Epitope 9	DKGKHKKAKKEKVKK	15	45.00% (9/20)	53.33	100.00
10	Epitope10	ILNKSFDSITGRLLN	15	55.00% (11/20)	20.00	100.00

Epitopes are present in all homologous sequences with a conservancy degree from 20 to 100 percent. Epitope 7 is the most abundant.

### Design and construction of multi-epitope vaccine candidate sequence

**Figure 1:**
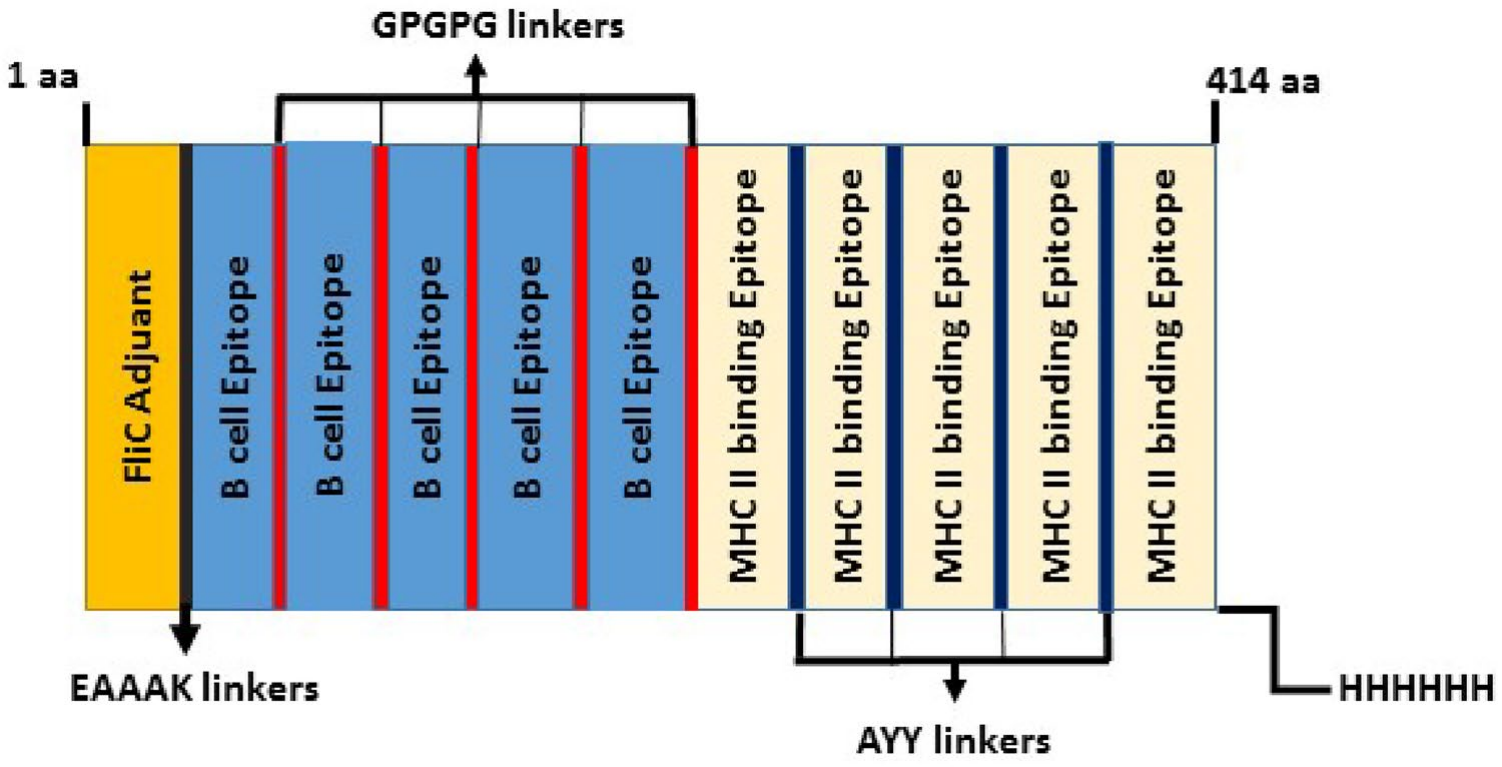
The schematic map of the final vaccine construct. In this construct, the adjuvant sequence was placed at the N-terminal and joined a multi-epitope sequence with the EAAAK linker (Black). GPGPG (Red) and AYY linkers (Blue) were used to bond B and HTL epitopes. The 414-amino acid long multi-epitope sequence was constructed. 6x-His-tag was placed at the C-terminal of the multi-epitope sequence to identify and purification aims.

In this step, selected high scored and affinity B-cell epitopes (a total of 5 linear B-cell epitopes) and MHC class II peptide-binding epitopes (a total of 5 epitopes) were joined together with GPGPG and AYY linker to generate the candidate sequence of a vaccine, respectively. Flagellin D0/D1 domains were added upstream of the above sequences as an adjuvant using the EAAAK linker to enhance the immunogenicity of vaccines. As well, six histidine residues codons were placed downstream of the sequence of the multi-epitope construct as a His-tag for the future purifying process. Therefore, the vaccine constructs with 414 amino acid residues were designed (Fig. 1).

### Physiochemical parameters and solubility prediction

The final protein's pI and Mw value predictions were 5.01 and 46 kDa, respectively. The half-life was considered to be 30 h in mammalian reticulocytes (in vitro), 20 h in yeast (in vivo), and 10 h in *E. coli* (in vivo). Aliphatic index and GRAVY were estimated at 65.12 and − 1.110, respectively. The instability index (II) was calculated with a 40.11 score. Upon expression, the constructed vaccine protein was soluble in the *E. coli* host with a solubility score of 0.759117.

### Antigenicity and allergenicity of the vaccine construct

The ANTIGENpro and VaxiJen 2.0 servers calculated the whole vaccine sequence antigenicity with the adjuvant sequence values of 0.92630 and 0.6976 with a threshold of 0.5 for the parasite model, respectively. The allergenicity of the vaccine was predicted using the AlgPred and AllerTOP v. two servers and resulted in non-allergenic.

### Secondary structure prediction

**Figure 2:**
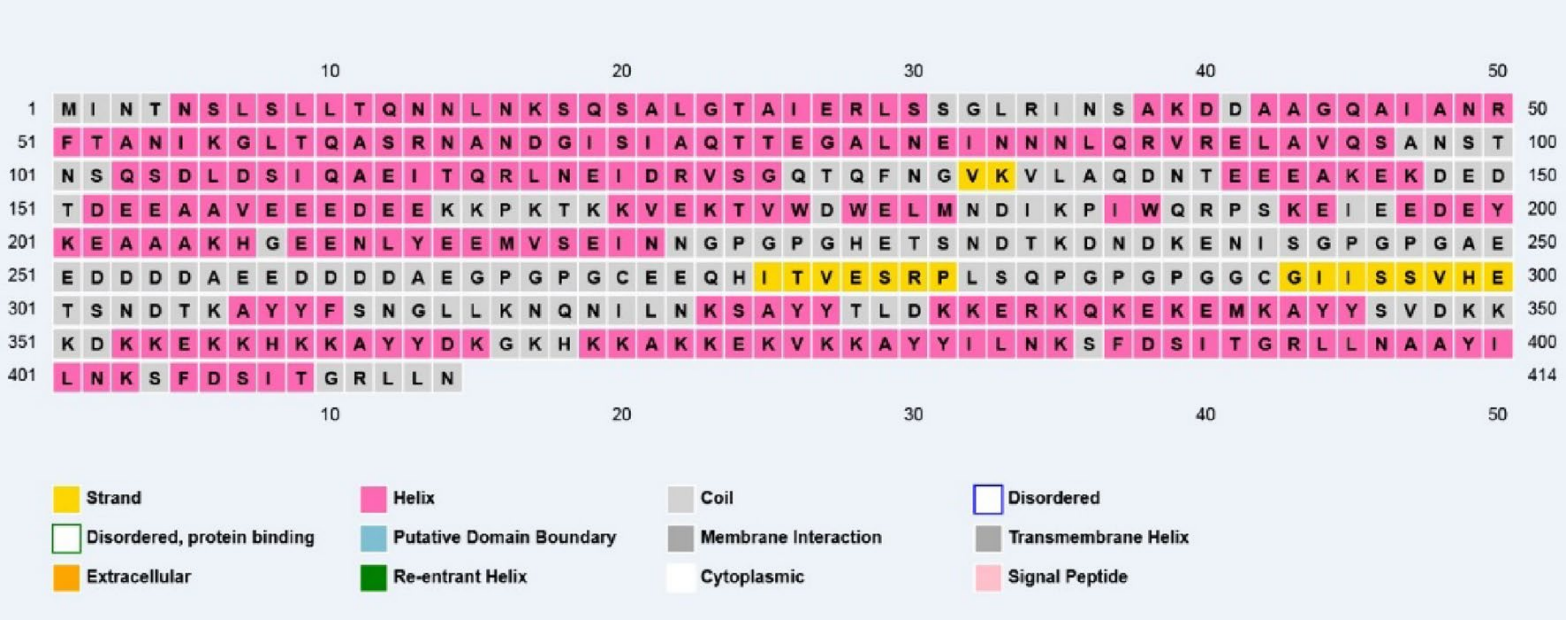
The PSIPRED server predicted a graphical demonstration of secondary structure properties of the final designed vaccine. Our vaccine's protein sequence comprised 60% alpha-helices, 39% beta strands, and 0.4% coils.

The predicted secondary structure using PSIPRED data was shown 60% alpha-helix, 39% beta-strand, and 0.4% coil in the final protein vaccine (Fig. 2). This secondary structure was used for refining the protein tertiary structure.

### 3D structure homology modeling and validation

**Figure 3:**
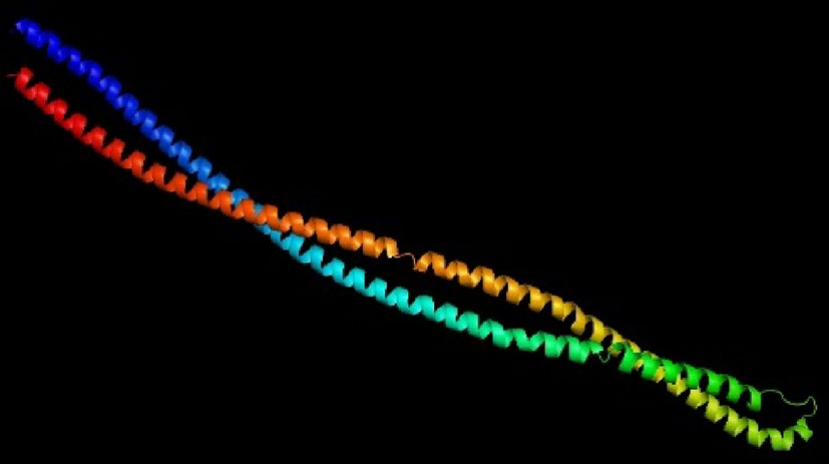
The 3D model of the final designed vaccine was obtained after homology modeling on Phyre2.

**Figure 4:**
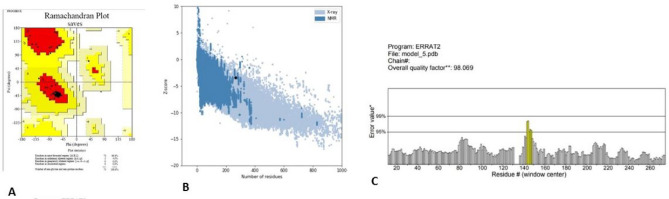
The validation of the final 3D model. (**A**) PROCHECK's Ramachandran plot illustrates that the residues are placed in the allowed (96%) and favored (4%) regions. (**B**) ProSA Z-score plot shows a -2.84 score in the range of conformation of the native protein. (**C**) ERRAT plot showed the overall quality factor to be 98.06%.

SWISS-MODEL, Phyre2, and I-TASSER are the servers used for 3D structure modeling. In this study, c2ch7A_11 model was selected from the Phyre2 server (Fig. 3) as the best model according to preliminary validation analysis. In the chosen model, analysis with PROCHECK's Ramachandran plot showed that 96% and 4% of residues are placed in favored regions and allowed, respectively (Fig. 4A). The ProSA z-score and ERRAT were − 2.84 and 98.06, respectively (Fig. 4B) and (Fig. 4C).

### Conformational (discontinuous) B-cell epitopes prediction

**Figure 5:**
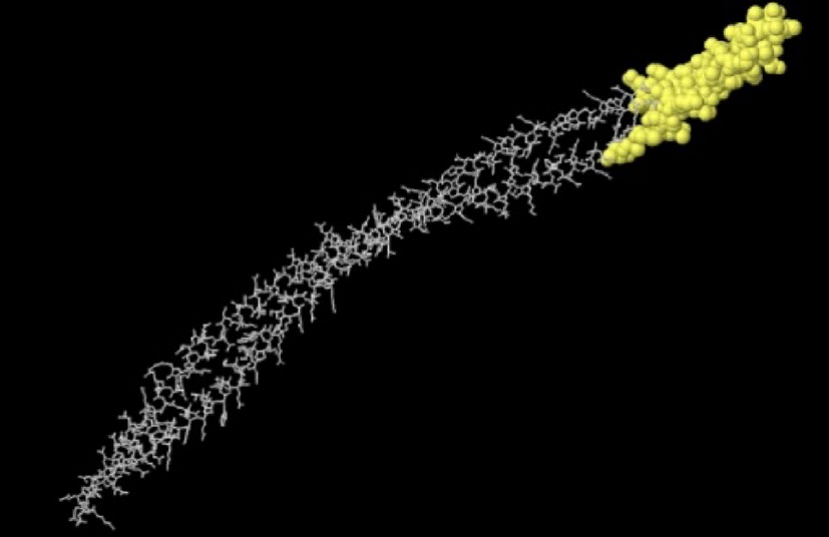
3D demonstration of the predicted conformational or discontinuous B-cell epitopes in the final designed vaccine construct. A yellow surface indicates the conformational or discontinuous B cell epitopes, and the rest of the residues is illustrated in grey sticks.

In the 3D model of the final designed vaccine construct, residues with a value of 0.7 or higher were identified as conformational epitopes (Table 4). Also, discontinuous epitopes predicted in the 3D structure of the final multi-epitope construct are shown (Fig. 5).

**Table 4:** Predicted conformational epitopes of the final designed vaccine by the ElliPro server.

No	Residues	Number of residues	Score
1	E143, :A144, :K145, :E146, :K147, :D148, :E149, :D150, :T151, :D152, :E153	11	0.934
2	:S8, :L9, :L10, :T11, :Q12, :N13, :N14, :L15, :N16, :K17, :S18, :Q19, :S20, :A21, :L22, :G23, :T24, :A25, :I26, :E27, :R28, :L29, :S30, :S31, :G32,:R34, :I35, :A38, :D41, :E257,:E258, :D260, :D261, :D262, :A263, :E264, :G265, :P266, :G267, :P268, :G269, :C270, :E271, :E272, :Q273, :H274, :I275, :T276, :V277, :E278, :S279	51	0.773
3	:E119, :R122, :G125, :Q126, :K133, :V134, :L135, :A136, :Q137, :D138, :N139, :T140, :E141, :E142, :E154, :A155, :A156, :V157, :E158, :E159, :E160, :D161, :E162, :E163, :K164, :K165, :P166, :K167, :T168, :K169, :V171, :E172, :V175, :W176	34	0.749

### Molecular docking of designed vaccine with TLR5

**Figure 6:**
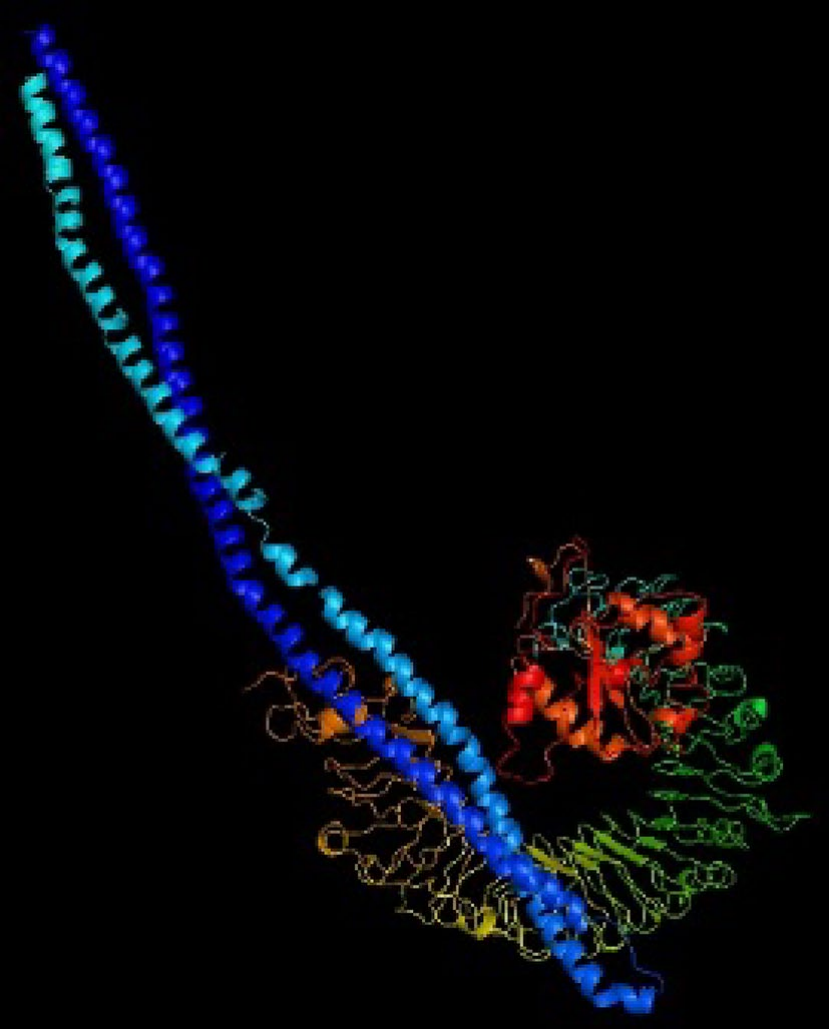
The docked model between the TLR5 receptor and the final designed vaccine using the ClusPro server. The vaccine protein is blue, and the rest of the residues are the TLR5 receptor. The lowest energy score of this docked model is − 1353.6, showing good binding affinity.

Molecular docking between the final construct model and TLR5 was done using the ClusPro server. The best possible docked complex with the highest binding affinity and total free energy (− 1353.6) was selected. The best docking between the final construct model and the TLR5 complex is shown in Fig. 6. The PyMOL 1.1eval server performs visualization and analysis of the docked complex in the next step.

### Protein–protein complex interfaces

**Figure 7:**
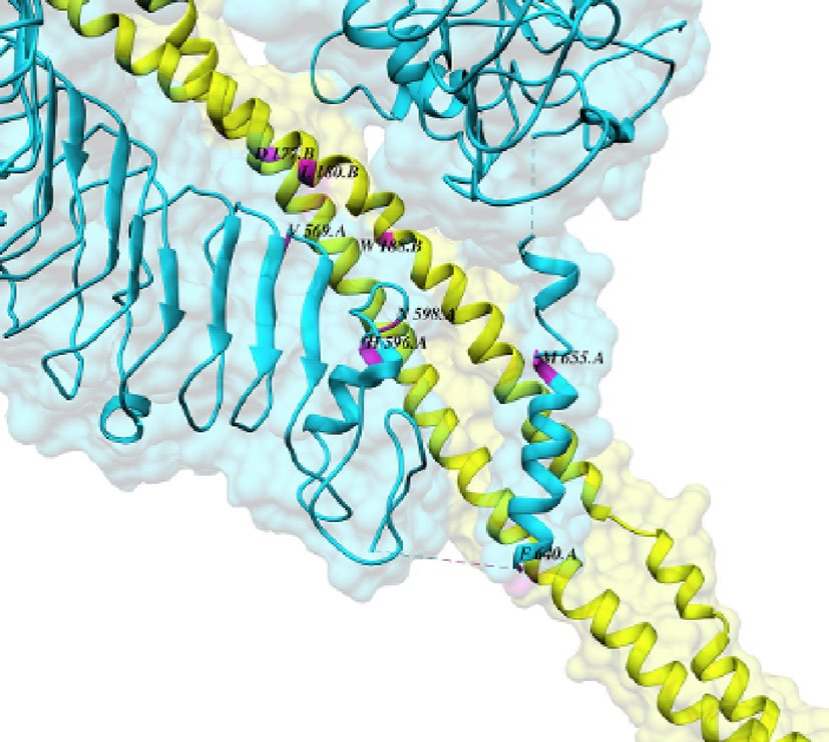
The interaction site of the designed vaccine and toll-like receptor 5 (TLR5) was analyzed by the PPCheck tool. The TLR5 is represented as cyan ribbons (chain A), the designed vaccine is illustrated as green color (chain B), and the interacting residues are in magenta. The residues are labeled, indicating the actual residue number of the structures and the relative chains.

To clarify the interacting residues at the interface of the protein–protein complex (TLR5 and designed vaccine), intra-structure hydrogen bonds, van der Waals interactions, and electrostatic interactions were defined. The hotspot residues are represented in Table 5. Figure 7 shows the site of interaction between two proteins in the protein complex and defines the hotspot residues.

**Table 5:** The interaction site between two proteins (TLR5 and designed vaccine) is defined as the hotspot residues in the protein complex.

Residue number	Residue name	Chain
569	VAL	A (TLR5)
598	ASN	A
596	HIS	A
180	LEU	B (designed vaccine)
640	PHE	A
178	TRP	B
177	ASP	B
655	MET	A
188	TRP	B

The hotspot residues are represented.

### Molecular dynamics simulation

**Figure 8:**
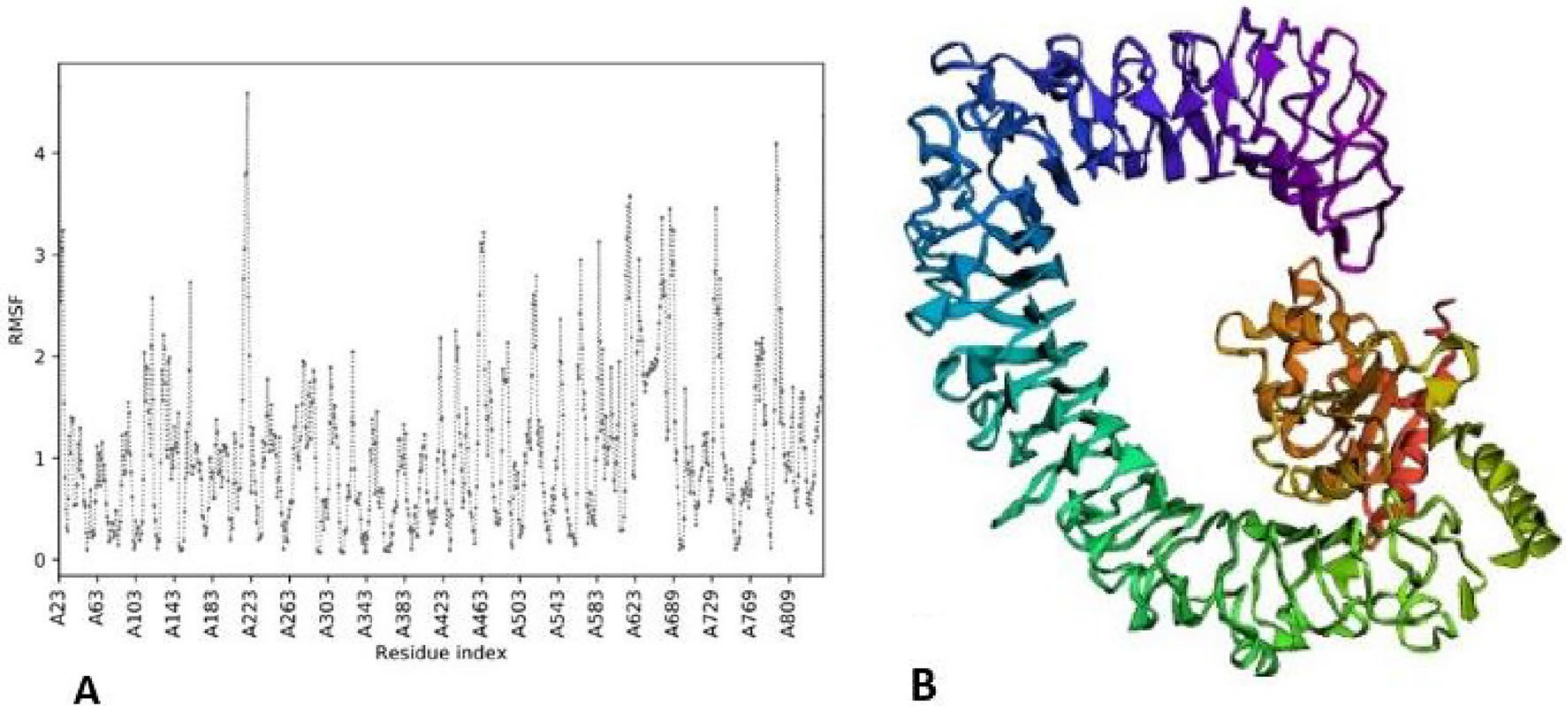
The molecular dynamics simulation of the vaccine-TLR5 docked complex. (**A**) The Root Mean Square Fluctuation (RMSF) plot of the protein vaccine structure demonstrates the fluctuations of MEV residues during the fast simulations. High degree fluctuation was identified in the residues at positions 219 and 617 in 4.58 Å and 3.57 Å. (**B**). The identified stable protein vaccine structure with fast flexibility simulations using the CABS-Flex server.

The molecular dynamics of our docked complex were done by CABS- Flex 2.0. The flexibility of the final docked complex has been analyzed at a 1.4 °C temperature and 50 cycles’ simulation for the 10 ns. We selected the first model as a stable protein vaccine structure based on the above parameters. The selected docked complex had the highest fluctuations levels in the residues (in chain A) at positions 219 and 617 in values of 4.58 Å and 3.57 Å, respectively (Fig. 8A). The stable protein vaccine structure using Cabs-Flex 2.0 (Fig. 8B).

### In silico cloning and codon optimization of vaccine construct

**Figure 9:**
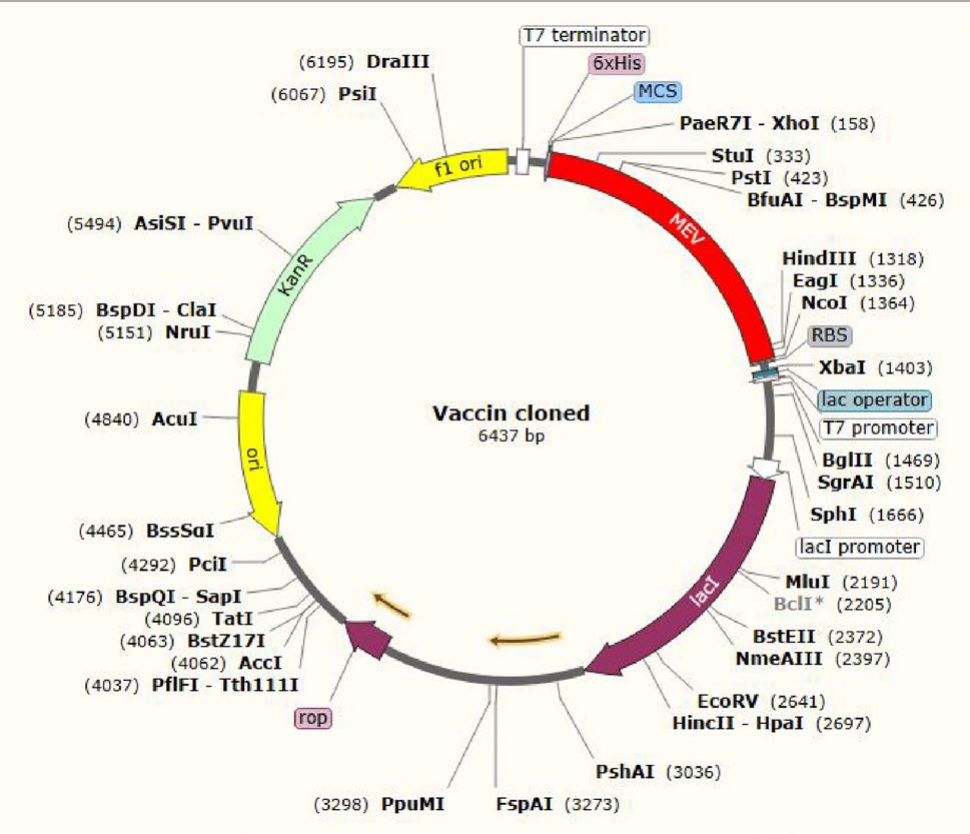
In silico cloning of multi-epitopes vaccine sequence into pET28a (+) expression vector using SnapGene software free-trial (https://www.snapgene.com/free-trial/), the red and gray semicircles represent the multi-epitopes vaccine sequence and the pET28a (+) backbone, respectively.

For efficient vaccine protein expression in the E. coli host, nucleotide vaccine sequence was reverse translated and codon-optimized using the GenScript tool. Some parameters such as GC content and codon adaptive index (CAI) for the optimized vaccine nucleotide sequence were respectively 46.32% and 0.85. These parameters were considered a good adaptation because they provided a high expression rate in *E. coli* K12*.* Finally, using *NcoI* and *XhoI* restriction sites, 1242 nucleotides as the optimized sequence was cloned into the pET28a vector. The 6xHis-tag at the C-terminal of the multi-epitope protein vaccine was placed for the purification process (Fig. 9).

### In silico immune responses simulation against the designed Vaccine

**Figure 10:**
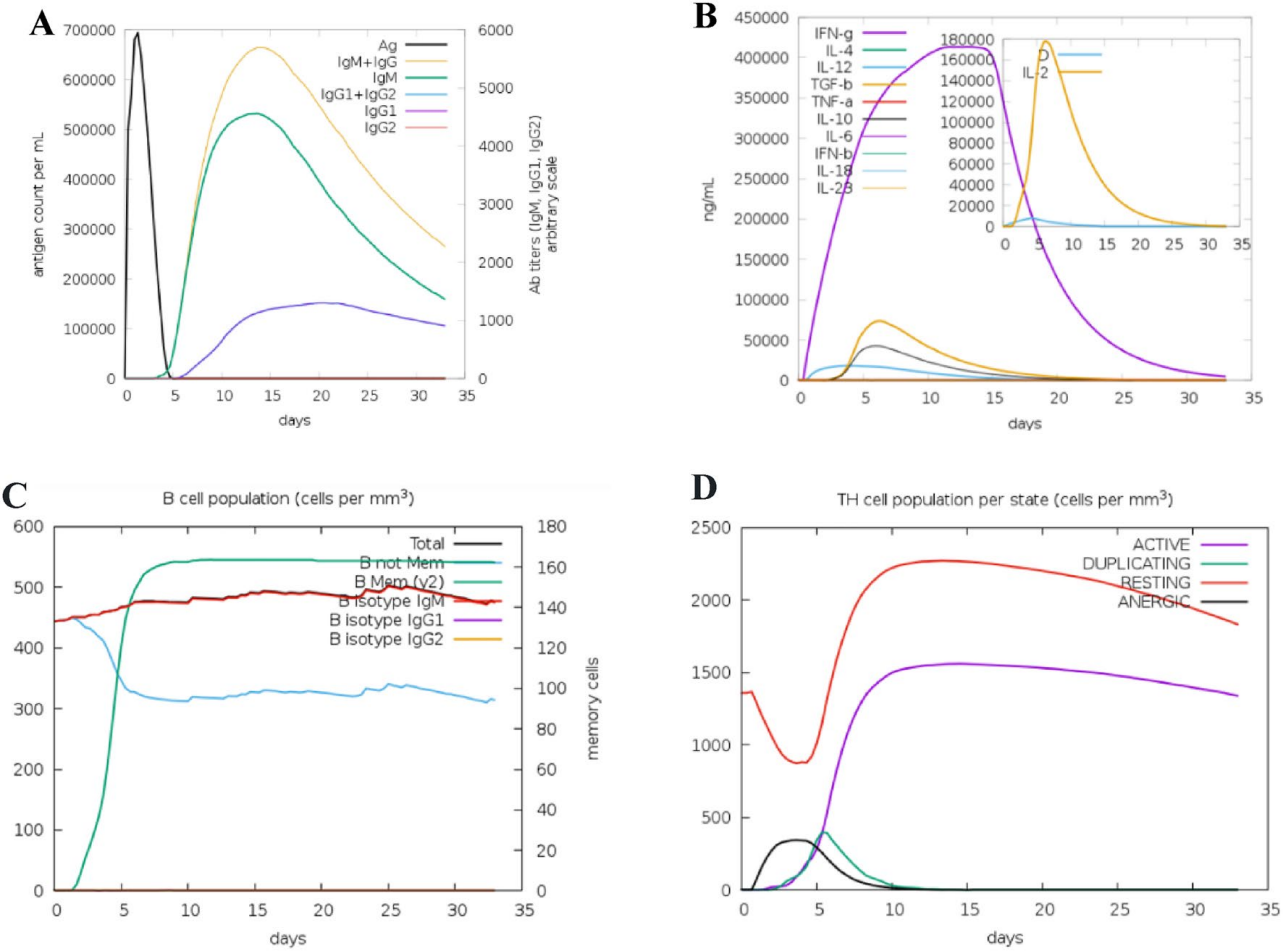
In silico immune simulation results of the designed vaccine from C-ImmSim server. (**A**) The titer of immunoglobulins was produced after the injection of the designed vaccine. (**B**) High titers of IFN-g and IL-2 were induced after vaccine administration. (**C**) B-cell populations prediction with a significant increase in the memory, non-memory cells, and IgM isotype. (**D**) The T-helper cell population per state (cells per mm3) levels increased after the injection.

The immune responses profile of the designed vaccine is shown in Fig. 10A. The combined IgM + IgG titer remained at about 680,000 xx/mL; the IgM titer alone was calculated to be around 530,000 xx/mL, and the combined IgG1 + IgG2 titer was about 150,000 xx/mL. These data show that the titer of immunoglobulins increased after the injection of the designed vaccine (as antigen) with a marked decrease in the antigen concentration. Analysis of interleukins (IL) and cytokines production showed high titers of IFN-g and IL-2, indicating that the antigen (designed vaccine) could trigger a strong and stable response. (Fig. 10B). B-cell populations with a significant increase in the memory, non-memory cells, and IgM isotype were predicted (Fig. 10C). The T-helper cell population per state (cells per mm^3^) represents increased levels after the injection (Fig. 10D).

## Materials and methods

### Retrieving of PfGARP and flagellin protein sequences

In this research, *P. falciparum* glutamic acid-rich protein (PfGARP) (P13816) (UniProt database at http://www.uniprot.org/) was selected as a parasite antigen. It then was evaluated for in silico study for potential B cell and MHC class II peptide binding prediction. A sequence of flagellin (fliC) protein (UniProt ID: Q8IFM5) (an agonist of TLR5) from Salmonella typhimurium was chosen as an adjuvant to enhance the efficacy of the vaccine^24^.

### Immuno-informatics analysis

#### B-cell epitope prediction

B-cell epitopes are potential antigens that are recognized by surface receptors of B-cell lymphocytes. The identification of these epitopes leads to producing a specific humoral response. Therefore, B-cell epitopes play a crucial role in vaccine efficiency^25^. In this step, the ABCpred (http://crdd.osdd.net/raghava/abcpred/) server as a sequence-based tool was used for predicting flexible length linear B-cell epitopes in PfGARP protein. This online web server uses different kernel methods to permit users to select the prediction method^26^.

#### MHC class II peptide-binding epitope (CD4+ T cell epitope) prediction

One of the critical purposes of immunology studies and vaccine development is identifying peptide epitopes restricted to MHC class II. The PfGARP protein amino acid sequence was submitted to the IEDB MHCII web server at http://tools.iedb.org/mhcii/ to predict MHC class II peptide binding epitopes. This server uses an artificial neural networks algorithm and classifies strong and weak binders to each HLA (HLADR, HLA-DP, and HLA-DQ) allele based on the affinity of peptides (in nM) and their % rank. In a way, a peptide with a lower percentile rank has a greater affinity^27^. This study selected epitopes with 15-mer lengths related to HLA-DR supertype alleles.

### Conservancy analysis of epitopes

The reference sequence of the glutamic acid-rich protein of *P. falciparum* served as a query for collecting homologous sequences by a BLAST search against a non-redundant protein database. The search was limited to Plasmodium (taxid:5820). The resulting collection of homologous sequences was used to investigate the conservancy level of each predicted epitope at IEDB (Immune Epitope Database)^28^ using the epitope conservancy analysis tool (http://tools.iedb.org/conservancy).

### Multi-epitope vaccine candidate construction

To generate the multi-epitope vaccine construct, the selected candidate epitopes, including 5 linear B-cell epitopes linked to each other using GPGPG and a total 5 high-affinity HTLs epitopes linked together AYY linkers, respectively. As an adjuvant, the fliC sequence was bound in the N-terminal region of the vaccine construct with the EAAAK linker.

### Prediction of antigenicity of the designed vaccine construct

Two servers, including Vaxijen 2.0 and ANTIGENpro, were used to study the antigenicity of the above vaccine construct. Vaxijen 2.0 is a free online available server at the address of http://www.jenner.ac.uk/VaxiJen. The basis of this server is the auto- and cross-covariance (ACC) transformation of sequences of target protein into the same amino acid sequence vectors. The VaxiJen v2.0 prediction method is alignment-free with the threshold value of 0.5 based on various protein physicochemical characteristics^29^. Also, ANTIGENpro is the alignment-free, pathogen-independent, and sequence-based predictor available at http://scratch.proteomics.ics.uci.edu. This server applies microarray data of protein antigenicity and shows an antigenicity index^30^. The ANTIGENpro server's accuracy, based on combined dataset and cross-validation experiments, was assessed at about 76%.

### Allergenicity prediction of designed vaccine construct

The AllerTOP V2.0 and Algpred servers were used to predict the allergenicity ability of the vaccine construct. AllerTOP V2.0 server is available at http://www.ddg-pharmfac.net/AllerTOP and is based on various methods such as auto- and cross-covariance transformation, k nearest neighbors (kNN) machine learning, and amino acid E-descriptors for classifying allergens^31^. At fivefold cross-validation, the accuracy of this server has been presented 85.3%. Algpred server is also available at http://webs.iiitd.edu.in/raghava/algpred and predicts allergenicity based on known epitope similarity in all regions of the target proteins with high accuracy^32^. In this study, among various integrated approaches in Algpred, the hybrid approach with a combined method (MAST + ARPs BLAST + IgE epitope + SVMc) was chosen for allergen prediction.

### Structural analysis

#### Different physicochemical properties and solubility analyses of vaccine construct

Different physicochemical properties of vaccine construct such as theoretical pI, stability profiling, molecular weight, amino acid composition, half-life, instability Index, aliphatic Index, and Average grand hydropathy were evaluated using ProtParam online server at the http://web.expasy.org/protparam/^33^. SOLpro is a scratch protein predictor that analyzed the solubility ability of vaccine construct with corresponding probability (≥ 0.5) and is available at http://scratch.proteomics.ics.uci.edu^34^.

#### Secondary structure prediction of vaccine construct

The vaccine constructs secondary structure was predicted with PSIPRED web free online server at the http://bioinf.cs.ucl.ac.uk/psipred/. The basis of this server is a highly accurate primary amino acid sequence that uses a stringent cross-validation approach to analyze the efficiency of the method. PSIPRED 3.2 servers can evaluate the obtaining output from PSI-BLAST (Position-Specific Iterated-BLAST) by combining two feed-forward neural networks^35^.

#### Tertiary structure prediction

Various servers, including SWISS-MODEL, I-TASSER, and Phyre2 were used for 3-dimensional structure modeling. Finally, based on the primary data, Phyre2 tool was chosen for the 3D structure modeling of the final vaccine construct. The Phyre2 (at http://www.sbg.bio.ic.ac.uk/phyre2) builds 3D models by advanced remote homology detection methods for a user’s protein sequence^36^.

#### 3D structure validation

After predicting 3D models, their performance and accuracy were checked with model validation tools. Some tools such as ProSA-web (https://prosa.services.came.sbg.ac.at/prosa.php)^37^, ERRAT (http://nihserver.mbi.ucla.edu/ ERRATv2/)^38^ and PROCHECK's Ramachandran plot analysis (https://servicesn.mbi.ucla.edu/PROCHECK/)^39^ existed for model validation. The ProSA program is frequently utilized for the validation of 3D protein structures. This program obtained its easy-to-use interface from ProSA-web. ProSA-web calculates the overall quality of the specific input 3D structures and demonstrates them as the z-score of experimentally PDB identified structures in a plot. ERRAT program works based on the statistics of non-bonded interaction between atoms in the described structure and is applied to verify crystallography identified protein structures. PROCHECK's Ramachandran plot program evaluates the protein structure stereochemical quality by analyzing overall structure geometry and residue-by-residue geometry.

#### Discontinuous B-cell epitope prediction in final MEV construct

Conformational epitopes play a major role in the antigen–antibody response^40^. The ElliPro server at the address http://tools.iedb.org/ellipro/ was employed on the validated 3D structure to predict conformational B-cell epitopes. ElliPro, a new web tool, predicts antibody epitopes according to protein structure geometrical properties and assigns a score as a Protrusion Index (PI) value averaged to each output epitope over epitope residues^41^. In the ElliPro method, the shape of the 3D protein structure is estimated by several ellipsoids. The ellipsoids with PI = 0.9 mean 90% and 10% of the protein residues are inside and outside the ellipsoid, respectively.

#### Molecular docking of designed vaccine with TLR-5 receptor

In this step, ClusPro 2.0 as a web-based server at the address https://cluspro.org was used to evaluate interaction patterns between the final vaccine construct and TLR5 receptor (PDB ID: 3J0A). This server performs the direct docking in 3 computational steps: (i) rigid-body docking, (ii) clustering of the structures based on the 1000 lowest RMSD value, and (iii) energy minimization and steric clashes removal^42^.

### Protein–protein interface analysis

The non-covalent bonds at the interface of the protein complex (TLR5 as receptor and designed vaccine as a ligand) were defined (as hotspots) by PPCheck^43^ tool is provided by http://caps.ncbs.res.in.

### Molecular dynamics (MD) simulations

To comprehend the biological functions of protein structures, recognizing flexible regions of protein structures is crucial. As an efficient alternative approach to conventional all-atom molecular dynamics (MD), CABS-flex has been developed for predicting protein structure fluctuations from a single protein model. CABS-flex 2.0 available at http://biocomp.chem.uw.edu.pl/CABSflex2. This web server predicts protein dynamics and structure using a CABS coarse-grained protein model^44^. A protein structure in the PDB format (or a protein PDB code) used as an input file and output set of all-atom models is obtained through trajectory clustering (by k-medoids method) and subsequent multi-step reconstruction and optimization approaches (using the Modeller package). The CABS-Flex 2.0 software provides ten alternative models based on optimum free energy, structural heterogeneity, and extremely stable configuration. Therefore, molecular dynamics simulations were done using the CABS Flex 2.0 server. The study applied the chosen docked TLR5-vaccine construct complex as the input data for a quick flexibility simulation under default setting of restraint parameters such as 1.4 °C temperature and 50 cycles’ simulation for the 10 ns. Besides protein flexibility, the contact map and root-mean-square fluctuations (RMSFs) of the atoms in the protein complex are measured in this server. All amino acid residues RMSF simulation in input protein is calculated in a nanosecond time by the CABS-flex server. The highest and lowest RMSF values indicate more flexibility and the limited motion of the system during the simulation process, respectively.

### In silico cloning and codon optimization of MEV construct

To express the vaccine construct in an appropriate expression vector, the GenScript Rare Codon Analysis in the address https://www.genscript.com/tools/rare-codon-analysis was applied for the study of the vaccine sequence codon optimization and reverse translation. The *E. coli* was selected as a host to express the vaccine sequence in the study. Two output indexes for high-level protein expression, including the percentage GC content and the codon adaptation index (CAI), were analyzed. The optimal CAI score is 1.0, although a score > 0.8 is also good. The optimal GC content can vary between 30 and 70%. These values usually are desired effects on transcriptional and translational efficiencies. Also, for cloning of final vaccine construct in pET-28a (+) vector, two Identification and cutting sites for *NcoI* and *XhoI* restriction site were added in N and C-terminals of cloned sequence, respectively. Then the cloning of the final vaccine constructs in the pET-28a (+) vector was performed in the SnapGene tool.

### In silico immune simulation against the designed vaccine

An agent-based modeling server C-ImmSim http://150.146.2.1/C-IMMSIM/index.php was used to predict the relationships between the human immune system and the foreign particle for observing the human immune response to the invading particle. The C-ImmSim is an agent-based immune simulator that uses the Position-Specific Scoring Metrix (PSSM) method to calculate the production of cytokines and other substances such as interferon and antibodies. C-ImmSim simulates the immunological response by combining the amino acid sequence of antigenic epitopes with lymphocyte receptors^45^. The vaccine construct was uploaded to the C-ImmSim web server with all parameters set to the defaults.

## Discussion

Despite effective treatments, *P. falciparum*, the most lethal form of malaria, is a global health and economic concern. This is related to an increase in *P. falciparum's* resistance to first-line antimalarial medicines^5,46^. Each cycle in the RBC lasts 2–3 days, but the trophozoite remains undetectable since it is concealed within the cell. The trophozoite exports antigens to the RBC membrane's outer surface, and these antigens could be identified^47^. People infected with Plasmodium have enough opportunity to build antibodies to the proteins that Plasmodium uses to infect RBCs^48,49^. PfGARP is found on the outer surface of the RBC membrane in trophozoite-infected RBCs. Its early expression in the trophozoite stage makes it an appropriate candidate for a *P. falciparum* blood-stage vaccine. It enables the killing of infected red blood cells before the parasite multiplies, thereby reducing the total parasite burden in a human host and the severity of malaria illness^7^. In the absence of immune effector cells or complements, Anti-PfGARP inhibits parasite growth by blocking and destroying trophozoite-infected RBCs^12^. The excellent news for malaria researchers is that the gene encoding PfGARP has slight genetic variation^9^. This factor has plagued malaria vaccine development since the beginning and led to the failure of several initiatives. Generally, vaccine development and production are often lengthy, challenging, and costly. Recently, researchers can benefit from in silico methodologies for the rational design of vaccines, particularly for pathogens, because of advancements in molecular immunology, the identification of immune-dominant epitopes, and progress in bioinformatics and immune informatics approaches^50^. Different bioinformatics tools and databases have recently emerged as the most cost-effective, rapid, and reliable method for predicting most antigenic regions of protein as potential targets for the advancement of subunit vaccines^51–54^. In a study using an immunoinformatics approach, Jelínková, Lucie et al. designed a novel and potent epitope-based malaria vaccine targeting the junctional region of the circumsporozoite protein, and as a result, the CIS43 VLP vaccine was introduced as a promising pre-erythrocytic malaria vaccine candidate^55^. Accordingly, we use bioinformatics methods in the present study to design a potential candidate epitope-based vaccine against malaria based on the PfGARP protein. For this purpose, we first selected PfGARP as the target antigenic protein for further analysis. T helper cells, which are necessary for almost all adaptive immune responses, are the principal mediators of cell-mediated immunity. They aid in activating B cells, which release antibodies and macrophages, and cytotoxic T lymphocytes, which kill infected target cells^56^. Conservancy analysis of epitopes was performed using the epitope conservancy analysis tool IEDB (Immune Epitope Database).

Additionally, in the following section, we predicted probable B-cell and T helper cell epitopes from the PfGARP protein to develop a multi-epitope vaccine (MEV) capable of inducing a humoral response. Then, to generate the MEV, the predicted epitopes (B-cell and HTL epitopes) were fused using suitable linkers (AAY and GPGPG linkers) as specialized spacer sequences. The AAY linkers play a role in increasing epitope presentation and removing junctional epitopes. In the context of GPGPG linkers, these linkers can stimulate T-helper responses and the conformationally-dependent immunogenicity of helper and antibody epitopes^57–59^. In this study, to compensate for these vaccines' low immunogenicity, the D0/D1 domains of the flagellin protein from S. Typhimurium bacteria were utilized as an adjuvant to boost the effectiveness of immune system stimulation^60^. Also, The EAAAK linker was employed to connect the D0/D1 domains at the N-terminal region of the multi-epitope sequence**.** For effectively separating the adjuvant interference with protein segments, the EAAAK linker helps to reduce interruption and increases the degree of expression and bioactivity of the target fusion protein. Finally, a candidate vaccine with a length of 414 amino acids, including some linear B-cell and HTL epitopes fused to the adjuvant sequence, was constructed. MEV was predicted to be antigenic with the probability of antigenicity 0.926395 and non-allergenic. Our MEV potentially produces a strong immune response without an allergic reaction, making it a potent vaccine. The structure's physicochemical properties were examined: The MEV construct has a molecular weight of 46 kDa. MEV has a theoretical pI value of 5.03, indicating that it is acidic. An extinction coefficient index for a chemical can be explained as the amount of light absorbed at a specific wavelength. The build has an extinction coefficient of 35995 M^−1^ cm^−1^. The candidate vaccine protein had an instability index (II) value of 40.11, indicating that our protein construct is relatively a stable protein (II > 40 implies instability). The aliphatic index measures how much space aliphatic amino acids (alanine, valine, isoleucine, and leucine) take up in the side chains of proteins. It could be viewed as a beneficial factor in increasing globular protein thermostability. The construct had an aliphatic index of 65.12, indicating that it was a thermostable protein. The GRAVY (Grand Average of Hydropathy) value for a peptide or protein is calculated by adding the sum of the hydropathy values of all amino acids by the number of residues in the sequence. Positive and negative values, respectively, represent the hydrophobic and hydrophilic qualities of a substance. The GRAVY value of our suggested construct was − 1.110, indicating that it is a hydrophilic protein^61^. The half-life is a forecast of how long it takes for half of the protein in a cell to vanish after production. ProtParam tool predicted the half-life of our construct in the following; 30 h (mammalian reticulocytes, in vitro), > 20 h (yeast, in vivo), and > 10 h (Escherichia coli, in vivo). For many biochemical and functional evaluations of recombinant proteins, the solubility overexpression in the *E. coli* host is one of the requirements that help in the efficient purification process in later stages^62^. The solubility of our multi-epitope protein construct is predicted with a probability of 0.759117 (by SOLpro service), indicating that overexpression of our multi-epitope protein in E. coli results in an insoluble state.

The PSIPRED method was used to analyze the secondary structure of our protein construct, which revealed that it mainly was alpha-helical (60 percent), 39 percent coils, and 0.4 percent of the amino acids in strand formation. Various servers got the vaccine constructs tertiary structure (SWISS-MODEL, phyre2, and I-TASSER). ProSA-web, RAMPAGE, and ERRAT servers were used in the validation process to identify potential faults and improve the quality of the projected 3D model. Based on validation data, the selected model had a high validation score and did not require further refinement. According to the Ramachandran plot, most residues are favored (96.0%) and 4.0% in permitted regions. This proposal used the Cluspro server to test the immunological interaction between the designed MEV construct and the TLR5 receptor. Cluspro displayed hundreds of docked models evaluated based on the protein surface's hydrophobicity, geometry, and electrostatic complementarity. Between the hydrophobicity models, we choose the best possible docked model. As a result, the best-docked complex was selected as the docked structure with the lowest energy score (-1353.6). Also, interacting residues at the interface of the protein–protein complex (TLR5 and designed vaccine) were revealed using PPCheck tool.

In this research, CABS-Flex 2.0 software was used for MD simulation. CABS-Flex presents the stable arrangement of the TLR5-designed vaccine complex. The individual amino acid residue's root means square fluctuation (RMSF) values (using CABS Flex 2.0) were described. The highest and lowest RMSF values show that our complicated structure fluctuates more and less during the simulation process, respectively. The MEV's structure fluctuates, indicating its considerable flexibility and validating it as a suitable vaccine structure.

To achieve high-level production and translation efficacy of our multi-epitope protein in *E. coli*, we used a codon optimization technique (strain K12). The CAI value (0.85) and GC content (46.32%) data were obtained in this study, indicating that the protein vaccine may be expressed more strongly in the E. coli K-12 system^42,63^. The MEV sequence was included in the pET-28a vector, which efficiently and effectively encoded the MEV protein in E. coli cells.

Simulation of immune responses was done using C-ImmSim server. The immune simulation data showed that after injection of our designed vaccine, appropriate immune responses were induced including (s) a significant rise in the immunoglobulins, high titers of IFN-g and IL-2, and development of memory B-cells and T-helper cell population.

We urge that validation experiments containing in vitro and in vivo studies be undertaken in the future to develop our candidate vaccine against malaria, based on the findings of this study.

## Conclusion

In the present work, to design a protective antibody-inducing multi-epitope vaccine against the blood-stage of *P. falciparum* based on B cell and T- helper cell epitopes of PFGARP antigen of *P. falciparum.* The adjuvant flagellin (fliC) protein was also incorporated into the vaccine construct to improve immunogenicity and induce, enhance, and deviate/direct the best form of the humoral immune response against *P. falciparum* trophozoite surface antigens. One of the advantages of the multi-epitope vaccine method is that it can elicit both humoral and cellular immune responses, and antibodies are required for the ultimate clearance of malaria parasites from the host's blood. The flexibility of the recombinant vaccination method could be helpful in the initial selection of promising candidates from thousands of candidates and subsequently improve their design and formulations. Therefore, the designed vaccine can induce long-term protective humoral immunity against the blood stage of *P. falciparum*. Nevertheless, to confirm the functionality of this multi-epitope vaccine, in vivo and in vitro immunological experiments are required.
